# ATNT: an enhanced system for expression of polycistronic secondary metabolite gene clusters in *Aspergillus niger*

**DOI:** 10.1186/s40694-017-0042-1

**Published:** 2017-12-19

**Authors:** Elena Geib, Matthias Brock

**Affiliations:** Fungal Genetics and Biology, School of Life Sciences, University of Nottingham, University Park, Nottingham, NG7 2RD UK

**Keywords:** Asp-melanin, P2A, Polycistronic mRNA, Tet-on system, Doxycycline, Terrein biosynthetic gene cluster

## Abstract

**Background:**

Fungi are treasure chests for yet unexplored natural products. However, exploitation of their real potential remains difficult as a significant proportion of biosynthetic gene clusters appears silent under standard laboratory conditions. Therefore, elucidation of novel products requires gene activation or heterologous expression. For heterologous gene expression, we previously developed an expression platform in *Aspergillus niger* that is based on the transcriptional regulator TerR and its target promoter P*terA*.

**Results:**

In this study, we extended this system by regulating expression of *terR* by the doxycycline inducible Tet-on system. Reporter genes cloned under the control of the target promoter P*terA* remained silent in the absence of doxycycline, but were strongly expressed when doxycycline was added. Reporter quantification revealed that the coupled system results in about five times higher expression rates compared to gene expression under direct control of the Tet-on system. As production of secondary metabolites generally requires the expression of several biosynthetic genes, the suitability of the self-cleaving viral peptide sequence P2A was tested in this optimised expression system. P2A allowed polycistronic expression of genes required for Asp-melanin formation in combination with the gene coding for the red fluorescent protein tdTomato. Gene expression and Asp-melanin formation was prevented in the absence of doxycycline and strongly induced by addition of doxycycline. Fluorescence studies confirmed the correct subcellular localisation of the respective enzymes.

**Conclusion:**

This tightly regulated but strongly inducible expression system enables high level production of secondary metabolites most likely even those with toxic potential. Furthermore, this system is compatible with polycistronic gene expression and, thus, suitable for the discovery of novel natural products.

**Electronic supplementary material:**

The online version of this article (10.1186/s40694-017-0042-1) contains supplementary material, which is available to authorized users.

## Background

Genome mining has revealed that fungal genomes contain a large number of yet unexplored secondary metabolite biosynthetic gene clusters [1]. Due to next generation sequencing approaches the number of available fungal genomes is steadily increasing as can be seen from the growing number of genomes in the 1000 fungal genomes project [2]. Interestingly, even highly related fungal species contain at least a few unique secondary metabolite biosynthetic gene clusters [3] and it has frequently been observed that more than one metabolite is produced from a single biosynthetic gene cluster [4]. Therefore, the potential of producing metabolites with interesting pharmaceutical characteristics appears nearly unlimited. However, as secondary metabolites are frequently produced in response to distinct biotic or abiotic stress factors [5], a large number of the respective biosynthetic gene clusters remains silent under laboratory conditions and, thus, their products unexplored. To exploit the full potential of fungal secondary metabolite production different strategies have been applied [6, 7].

One approach that can be directly applied to cultivable fungal species is the addition of epigenetic modifiers [8] or co-cultivation with other microbes, which may result in the specific induction of biosynthetic gene clusters [9]. However, while this strategy may lead to the production of novel metabolites, a direct correlation between biosynthetic gene cluster and metabolite product remains difficult. Another strategy is the overexpression of a transcriptional regulator controlling a specific biosynthetic gene cluster [10]. Unfortunately, not all secondary metabolite biosynthetic gene clusters contain a transcriptional activator in direct proximity to their biosynthetic genes [11], which may hamper this approach. In addition, global transcriptional regulators may overrule the activation from a cluster specific transcription factor as shown for the dihydroisoflavipucine biosynthesis in *Aspergillus terreus* [12]. While this biosynthetic gene cluster contains a specific transcriptional activator that is indispensable for its activation, the activating effect is overruled in the presence of glucose through the carbon catabolite repressor CreA [12].

The strategy of targeted activation of cluster specific transcription factors additionally requires the ability for genetic modification of the natural producer strain and may not be suitable for many fungal species. Therefore, recent approaches used the generation of fungal artificial chromosomes (FAC) to clone and transfer whole fungal gene clusters into genetically amenable fungal expression platform strains [13]. In a previous study, 56 gene cluster containing FACs with yet uncharacterised biosynthetic genes from *Aspergillus wentii*, *Aspergillus aculeatus* and *A. terreus* were transferred to *Aspergillus nidulans*, which resulted in the identification of 17 novel metabolites from 15 different FACs [13]. However, not all gene clusters were successfully activated in the recombinant host, which may be due to the lack of transcriptional activators, repressing conditions or the lack of the correct starter metabolites in the heterologous host.

It has also been shown that induction of secondary metabolite biosynthetic gene clusters in a heterologous host can be achieved by regulating the expression of the global regulator of secondary metabolism LaeA in *Aspergillus* species [14]. In this respect, a transfer of the biosynthetic gene clusters for monacolin K from *Monascus pilosus* and terrequinone A from *A. nidulans* resulted in successful product formation after overexpression of *leaA* in *Aspergillus oryzae* [15]. However, induction of several biosynthetic gene clusters appears independent from LaeA control and a specific transcriptional activator in direct proximity to the biosynthetic gene cluster may be lacking. Therefore, a different strategy for gene activation was successfully applied to *A. nidulans*, in which a serial promoter exchange of each individual gene of a biosynthetic gene cluster was performed. This strategy resulted in the identification of the proteasome inhibitor fellutamide B and its resistance conferring gene *inpE* [16]. Although successful, this strategy required several rounds of metabolite screening, marker regeneration and subsequent transformation and appears prohibitively time consuming for routine applications.

Due to these challenges it remains difficult to recommend an expression system that allows for high throughput screening for all yet uncharacterised secondary metabolite biosynthetic gene clusters. Heterologous gene expression generally aims for high product yields to elucidate the structure of the metabolite and to analyse its biological activity. A prerequisite for this is the high level expression of target genes, which can be achieved by generating multiple copy integrations, selection of strong promoters or a combination of both [17, 18]. Recently, we introduced a heterologous expression system that uses an *Aspergillus niger* strain as expression platform that contains regulatory elements from *A. terreus* [18]. These regulatory elements consist of the terrein biosynthetic gene cluster specific transcriptional activator TerR and its target promoter P*terA*. When expression of *ter*R is controlled by the *A. oryzae* amylase promoter and a reporter gene is expressed under P*terA* control the induction level of the amylase promoter gets amplified through this coupled system [18]. In addition, a SM-Xpress vector has been constructed that allows easy generation of expression plasmids by in vitro recombination with the target gene. This expression system had been successfully applied for the identification of lecanoric acid as product from the *A. nidulans orsA* gene [18], has enabled the heterologous in vivo reconstruction of the *A. terreus* Asp-melanin biosynthetic pathway in *A. niger* [19] and was recently successfully used for identification of basidioferrin, which is a novel siderophore produced from a non-ribosomal peptide synthetase (NRPS) that is widely distributed among basidiomycetes [20].

Another challenge in heterologous expression of secondary metabolite biosynthetic gene clusters derives from possible toxicity of resulting metabolites. Therefore, a tight regulation of gene expression is favoured as it allows for the formation of fungal biomass prior to induction of the expression of target genes. In this respect a tuneable Tet-on/Tet-off expression system has been adapted for use in *Aspergillus* species [21, 22]. The Tet-on system uses a reverse tetracycline-controlled transactivator that enables titratable induction of gene expression by the addition of the tetracycline derivative doxycycline. In the absence of doxycycline gene expression remains at low background levels, but expression gets strongly induced by addition of doxycycline [21, 22].

As most secondary metabolites are produced from biosynthetic gene clusters, production of the final metabolite generally requires the heterologous expression of more than only one single gene. While a strategy of subsequent transformations with isolated genes accompanied by a marker recycling technique works for clusters comprising only a small number of genes, this procedure is extremely time consuming and probably not suitable for larger clusters containing five or more genes. Therefore, another strategy is the use of self-splicing viral peptide sequences such as the 2A peptide that separates proteins from a polycistronic messenger in different viruses such as the porcine teschovirus-1 (P2A). P2A and similar sequences have been successfully used to separate individual proteins in a range of different eukaryotic organisms [23, 24] among them yeasts such as *Saccharomyces cerevisiae* [25, 26] and *Pichia pastoris* [27]. A recent study also used a 2A peptide in *Trichoderma reesei*, in which the gene coding for the cellobiohydrolase Cel7A from *Penicillium funiculosum* was combined in a single transcript with the eGFP coding gene to ease screening of cellobiohydrolase positive transformants [28]. Importantly, this technique has also been applied for heterologous production of penicillin in the filamentous fungus *A. nidulans* by genetic engineering of a synthetic *Penicillium chrysogenum* penicillin biosynthetic gene cluster [29]. Despite low yields, penicillin K was successfully produced by *A. nidulans* transformants expressing the polycistronic penicillin biosynthetic gene cluster [29], indicating that this strategy is suitable for use in fungal secondary metabolite biosynthesis. The suitability of P2A was further confirmed in a recent study on enniatin biosynthesis in *A. niger*, in which two genes required for enniatin biosynthesis and a luciferase were separated by P2A sequences [30]. While a positioning effect in dependence of the gene order in the polycistronic messenger was observed, all strains produced enniatin and displayed light emission from luciferase activity. Positioning effects were also observed in the cellobiohydrolase expression in *T. reesei* [28] and murine cells [31], indicating that despite polycistronic gene expression the amount of individual proteins may vary depending on the gene order in the expression construct.

Here, we aimed to generate an optimised fungal heterologous expression system by combining the three latter aspects of heterologous secondary metabolite production in *A. niger*: (1) using the expression amplification system of TerR/P*terA* under (2) fine-tuneable control of the Tet-on system for expression of (3) polycistronic mRNA of the Asp-melanin biosynthetic genes combined with a fluorescent reporter to study correct subcellular localisation of enzymes.

## Results

### Integration of Tet-on control into the coupled TerR/PterA expression system

**Fig. 1:**
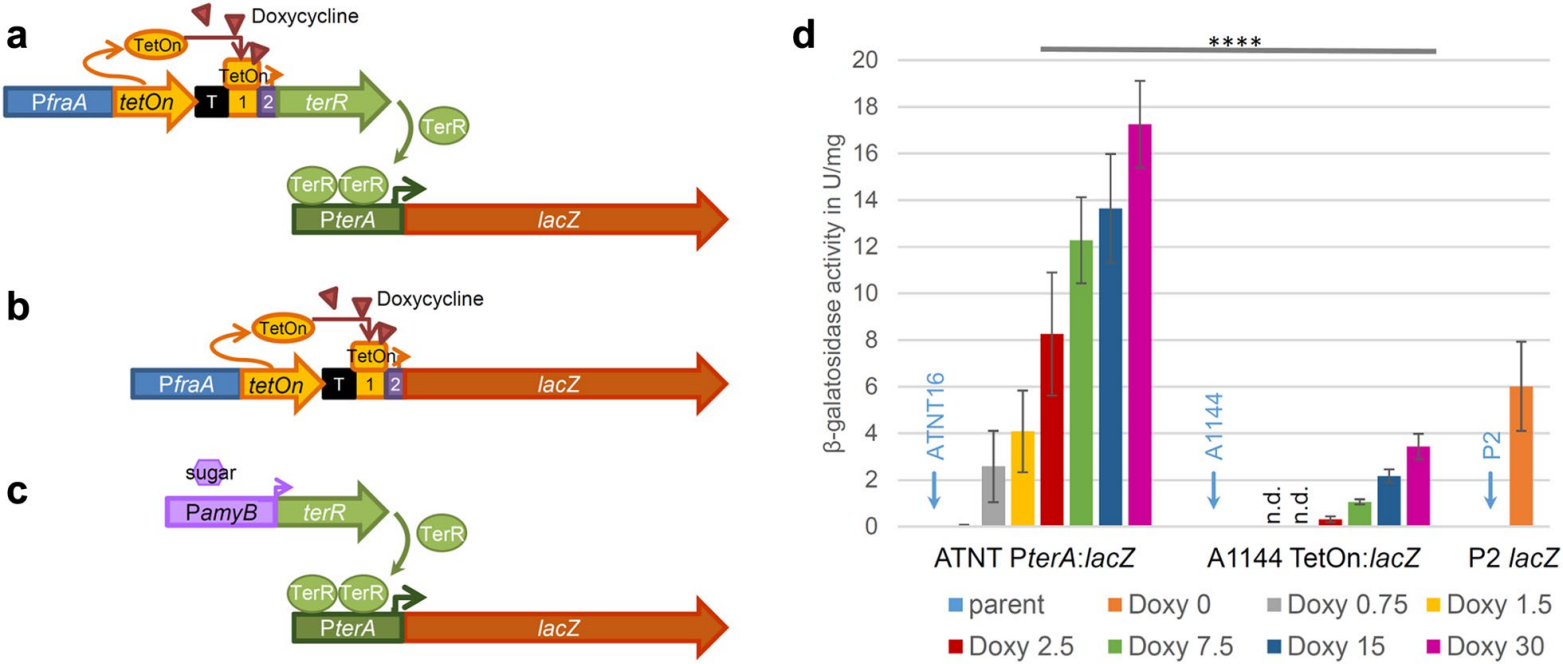
Analysis of β-galactosidase reporter activity from different expression systems. **a**–**c** Schematic representation of expression systems. **a** The ATNT system, in which expression of the transcriptional regulator *terR* is controlled by Tet-on. The reverse transactivator is constitutively expressed from the *fraA* promoter and transcription is terminated by the *ergA* terminator (T). In the presence of doxycycline the reverse transactivator binds to the *tet*-*on* responsive element (*1*), which leads to transcription of *terR* from the minimal *gpdA* promoter (*2*). TerR specifically recognises its P*terA* target promoter, which results in transcription of the β-galactosidase gene *lacZ*. **b** Direct control of *lacZ* expression by the Tet-on promoter system. **c** The P2 expression system. The *amyB* promoter controls expression of *terR* and is activated in the presence of sugars. TerR controls expression of *lacZ* by binding to the P*terA* promoter. **d** β-Galactosidase activity from expression systems shown in **a**–**c**. ATNT and Tet-on *lacZ* strains were grown in the presence of different doxycycline concentrations, whereas the P2 *lacZ* strain was cultivated without doxycycline. The positions of activities derived from parental strains are highlighted by arrows. Three individual transformants from each expression system were measured in biological and technical duplicates. Bars represent mean values of all measurements and standard deviations are indicated by error bars. Multiple t-tests using the Holm-Sidak method were applied, which confirmed significantly different expression levels in all groups in dependence of the doxycycline concentration. (****p < 0.0001; *n. d.* not determined)

We previously developed a heterologous expression system in *A. niger* that bases on the transcriptional activator TerR from the *A. terreus* terrein biosynthetic pathway and its *terA* (P*terA*) target promoter [18]. In this combination the induction level of P*terA* directly depends on the transcriptional level of the *terR* gene [18]. Furthermore, the activity of the promoter controlling *terR* expression gets amplified at the target promoter P*terA* as reporter expression in the coupled system was significantly higher than direct expression of reporter genes [18]. In this first version of the expression system, we controlled *terR* expression by either the glyceraldehyde-3-phosphate dehydrogenase promoter P*gpdA* or the amylase promoter P*amyB*. As both of these promoters derive from primary metabolism, their use may interfere with fungal metabolic physiology. In addition, both promoters are difficult to silence and P*amyB* shows significant background activity even when *A. niger* is grown on casamino acids in the absence of any sugars. As this background promoter activity may hamper the production of toxic metabolites, we replaced P*amyB* in the control of *terR* by the reverse tetracycline-controlled transactivator (Fig. 1a) containing the *fraA* promoter sequence for improved cassette stability. The *fraA* gene encodes a putative ribosomal subunit and had been identified from microarray analyses showing a similar expression pattern as the glyceraldehyde-3-phosphate dehydrogenase and is assumed to be constitutively expressed [22]. The Tet-on:*terR* construct was used for transformation of the *A. niger* A1144 strain (Fungal Genetics Stock Center, Kansas, USA) and resulting transformants were analysed for full length single copy integration into the genome (Additional file 1). The resulting expression platform strain ATNT16 (ATNT = **A**1144 **T**et-o**n**:***t*** *erR*) was analysed for its performance in gene expression.

### Analysis of β-galactosidase reporter gene expression

To elucidate the performance of the new Tet-on-controlled expression system in *A. niger* ATNT16, we generated β-galactosidase reporter strains. Two different constructs were made that both contained the *lacZ* gene from *Escherichia coli* as reporter. The first construct contained a fusion of P*terA* with the *lacZ* gene (P*terA:lacZ*) for transformation of the ATNT16 strain (Fig. 1a). The second construct contained a fusion of the Tet-on promoter system directly with the *lacZ* gene (Tet-on*:lacZ*) for transformation of the parental *A. niger* strain A1144 (Fig. 1b). This enabled the comparison of doxycycline dependent gene activation in the coupled amplification system of TerR/Pt*erA* under control of Tet-on against the direct reporter gene induction by the Tet-on system. After transformation of the respective *A. niger* strains, transformants with a single copy integration of the respective reporter construct were identified by Southern blot analysis (Additional file 2) and three independent transformants from each construct were selected for downstream investigation of reporter activities. In addition, three reporter strains from the original P*amyB*:*terR* expression platform (P2 strain; *terR* gene under control of the amylase promoter) with single copy integration of the *lacZ* gene under control of P*terA* [18] were included (Fig. 1c). This allowed comparison of expression properties of the new ATNT16 expression platform with that of the previous platform strain P2. All strains were cultivated for 24 h on 100 mM glucose containing minimal media with 20 mM glutamine as nitrogen source and 1% talc to avoid the formation of cell pellets [32]. For the Tet-on-containing strains parallel cultures were supplemented with various amounts of doxycycline in a range between 0 and 30 µg/ml. All strains were cultivated in two biological replicates and β-galactosidase activity was determined from cell-free extracts in technical duplicates (Fig. 1d). The average specific β-galactosidase activity of the P2 reporter strain on this glucose containing medium was about 6 U/mg, which was in agreement with previous determinations under this growth condition [18]. Both, the ATNT16 reporter strains as well as the Tet-on:*lacZ* strains only revealed very low background activity when cultivated in the absence of doxycycline (< 0.05 U/mg). Addition of doxycycline to the Tet-on:*lacZ* strains resulted in a titratable induction of reporter activity, reaching a maximum of 3.4 U/mg at 30 µg/ml of doxycycline. The ATNT16 strain with the P*terA*:*lacZ* reporter construct showed significant reporter activity of 2.5 U/mg already at 0.75 µg/ml, which further increased to 17.3 U/mg at 30 µg/ml of doxycycline. This latter activity is about five times higher than the maximum activity obtained from the uncoupled system at 30 µg/ml in which Tet-on directly induces the expression of the target gene. Thus, the ATNT16 expression platform with the *terR* gene under Tet-on control is tightly regulated in the absence of doxycycline and strongly induced by its addition. However, accompanied with high reporter gene expression, biomass formation in the presence of 30 µg/ml doxycycline in the ATNT16 reporter strains was significantly reduced. This may be due to the high reporter protein production during initiation of germination and seems independent from high levels of activated transactivator protein as the ATNT16 strain without *lacZ* reporter construct showed no growth defects in the presence of 30 µg/ml doxycycline and β-galactosidase background activity in the ATNT16 strain did not increase by the addition of different doxycycline concentrations (not shown).

### Production of aspulvinone E in the ATNT16 expression platform

**Fig. 2:**
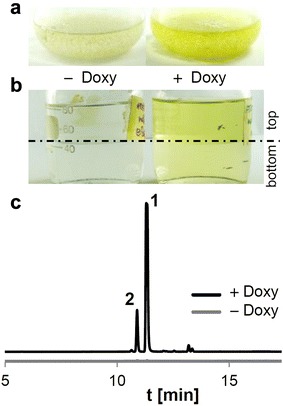
Heterologous expression of the aspulvinone E synthetase gene *melA* in the ATNT system. **a** Cultures of an ATNT16 *melA* ^OE^ strain cultivated with or without 15 µg/ml doxycycline and **b** ethyl acetate extraction of these cultures (top phase is ethyl acetate). **c** Extracts from (**b**) were dried and solved in 1 ml of methanol and 10 µl were subjected to HPLC analysis. The doxycycline induced culture confirms the biosynthesis of aspulvinone E (**1**) and its stereoisomer isoaspulvinone (**2**) as deduced from authentic standards

Our reporter gene analyses indicated that the Tet-on-controlled TerR/P*terA* system is tightly regulated and allows high level gene expression in the presence of doxycycline. To test whether this also transfers to secondary metabolite production, we used the *melA* gene, which encodes the aspulvinone E synthetase from *A. terreus* [19] under control of P*terA* and transferred this construct into the Tet-on:*terR* strain ATNT16. After selection for single copy integration (Additional file 3), strains were cultivated for 48 h in glucose minimal medium either in the absence or presence of 15 µg/ml doxycycline. In accordance with a light yellow colour of aspulvinone E, the cultures grown in the presence of doxycycline turned yellow (Fig. 2a) and the main proportion of the coloured substance solved in the ethyl acetate phase during extraction of culture filtrates (Fig. 2b). In contrast, no obvious yellow colouration of the culture or the ethyl acetate phase was observed in the control cultures without doxycycline (Fig. 2a, b). To confirm that aspulvinone E was produced only under inducing conditions samples were analysed by HPLC using reversed phase chromatography on a C_18_ column. As shown in Fig. 2c the induced culture revealed a strong signal for aspulvinone E and a minor signal of its stereoisomer isoaspulvinone E [19]. By contrast, only extremely weak background signals were detected in the control culture. These results are in agreement with the β-galactosidase reporter studies and confirm that (1) metabolite production is suppressed in the absence of doxycycline and (2) high yields of metabolites can be achieved under inducing conditions even in strains only carrying a single copy integration of the gene of interest.

### Model gene cluster expression from polycistronic mRNA using the P2A peptide

In the next step we aimed in the expression of multiple genes in the Tet-on-controlled TerR/P*terA* system by engineering polycistronic mRNAs. For a proof-of-concept, the Asp-melanin pathway combined with a fluorescent reporter was used [19]. Asp-melanin is the conidial pigment produced by *A. terreus* and is distinct from the dihydroxynaphtalene melanin found in conidia of other *Aspergillus* species. This melanin pigment does not derive from a naphtopyrone precursor that is produced by a polyketide synthase rather than an aspulvinone E synthetase, which is a non-ribosomal peptide synthetase-like (NRPS-like) protein [19]. This pigment biosynthesis pathway appeared most suitable as: (1) Asp-melanin is produced from only two proteins, which are the aspulvinone E synthetase MelA and the tyrosinase TyrP; (2) co-expression of individually controlled genes in the *A. niger* P2 strain resulted in brown mycelium due to the formation of Asp-melanin, which is easy to visualise; (3) Asp-melanin formation requires the correct subcellular localisation of both enzymes as MelA requires the reducing environment of the cytoplasm and TyrP the oxidising environment of Golgi or ER (4) protein localisation and cleavage efficiency can be visualised by using the red fluorescent protein tdTomato as a reporter.

**Fig. 3:**
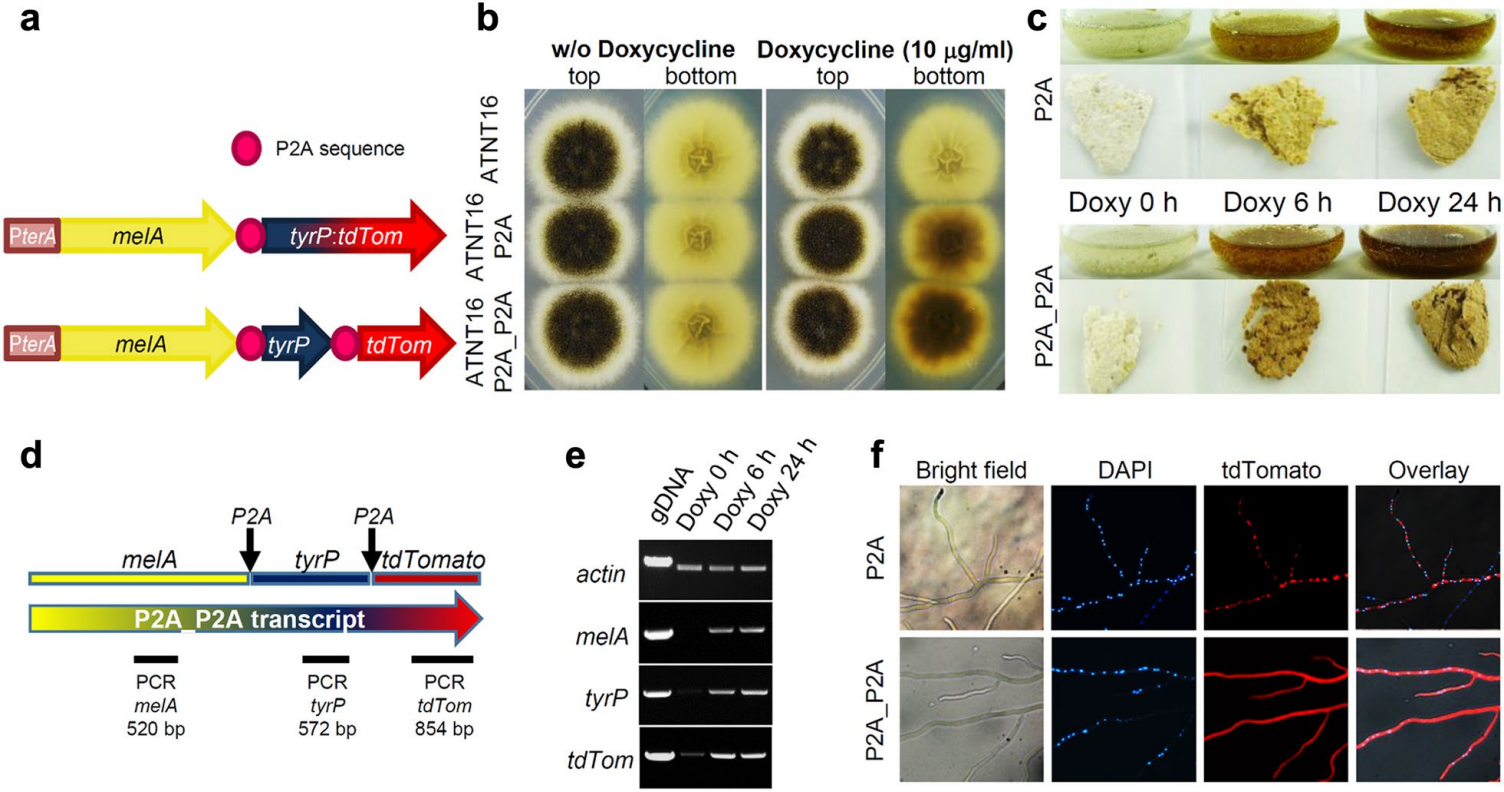
Asp-melanin formation and subcellular protein localisation from polycistronic gene expression in the ATNT system. **a** Schematic presentation of polycistronic expression constructs separated by P2A sequences. *tyrP:tdTom* denotes an in frame fusion of the *tyrP* gene with the gene coding for the red fluorescent protein tdTomato. **b** Colonies in top and bottom view of the parental strain ATNT16 and strains carrying the expression construct with one or two P2A separations grown in the absence and presence of doxycycline. Addition of doxycycline induces the formation of Asp-melanin, which is indicated by brown colouration of mycelium in the bottom view. **c** Liquid cultures of ATNT16 strains carrying the expression construct with one or two P2A sequences. Mycelium was harvested after 24 h of incubation. Cultures were grown without doxycycline (Doxy 0 h) or were induced with doxycycline for the last 6 h of total incubation time (Doxy 6 h) or for the whole 24 h (Doxy 24 h). A stronger colouration of mycelium is observed when TyrP and tdTomato are separated by an additional P2A peptide. **d** Scheme of the polycistronic P2A_P2A mRNA. Localisation of the individual gene sequences are indicated above and localisation and size of PCR products for verification of transcription are shown below the transcript. **e** Semiquantitative RT-PCR on cDNA derived from cultures in **c**. The actin gene was used for normalisation of cDNA. Amplification from genomic DNA (gDNA) is shown as a control with a decrease in fragment size of the actin gene due to intron splicing. Full length-transcription of the polycistronic messenger is confirmed by PCR products from all genes when grown in the presence of doxycycline. **f** Fluorescence analysis for subcellular localisation of proteins produced from the two polycistronic expression constructs. Nuclei are shown in blue by DAPI staining. Red fluorescence indicates localisation of tdTomato. In the P2A construct the fusion of TyrP with tdTomato reflects a punctuated fluorescence consistent with ER and Golgi. When tdTomato is separated by P2A in the P2A_P2A construct, tdTomato localises to the cytoplasm

For the separation of individual proteins during ribosomal translation, the 22 amino acid 2A peptide (P2A, GSGATNFSLLKQAGDVEENPGP) sequence from porcine teschovirus-1 was used [24], whereby codon sequences of individual P2A peptides were varied on DNA level to allow directed in vitro recombination into the SM-Xpress expression vector that contains the *terA* promoter, the *trpC* terminator sequence and a resistance gene for selection of transformants [18]. Two different polycistronic constructs consisting of the *melA* gene, the *tyrP* gene and the gene coding for tdTomato were generated to test the efficiency of P2A cleavage and protein localisation in the ATNT16 expression platform (Fig. 3a). For the first construct all three genes were separated by a P2A coding sequence (P2A_P2A construct), which was assumed to result in three individual functional proteins under inducing conditions that lead to brown mycelium and a cytoplasmic localisation of tdTomato as this reporter does not contain a subcellular localisation signal. The second construct only contained a single P2A sequence (P2A construct) separating the *melA* and *tyrP* genes, whereby the gene coding for tdTomato was fused in frame with the *tyrP* gene [19]. Here, we expected the formation of brown mycelium under inducing conditions, but a fluorescence localisation in subcellular organelles of ER and Golgi, which would confirm the correct targeting of TyrP. ATNT16 was transformed with the respective constructs and resulting transformants were analysed by Southern blot analysis (Additional file 4) for single copy integration. For analysis of selected transformants split plates were prepared with glucose minimal medium containing 0 or 10 µg/ml of doxycycline. On these plates the control strain ATNT16 as well as strains containing either the P2A or the P2A_P2A construct were spotted and pictures taken after 72 h of incubation. As shown in Fig. 3b, all strains showed similar growth and conidia formation in the top view of plates. However, the bottom view shows that mycelium of strains with the P2A and the P2A_P2A construct turned dark brown. Similarly, liquid cultures inoculated with conidia of the respective transformants were incubated for a total of 24 h in absence (Doxy 0 h) or presence of 15 µg/ml doxycycline. Thereby, the inducer doxycycline was added either directly at the start of cultivation (Doxy 24 h) or after a pre-cultivation for 18 h to allow for conidia germination and hyphae formation prior to induction resulting in an induction time of 6 h (Doxy 6 h). In the absence of doxycycline mycelia of cultures containing either of the two different constructs remained uncoloured, whereas mycelia turned brown under inducing conditions even when induced for only 6 h (Fig. 3c). Therefore, both constructs produce functional proteins that produce Asp-melanin and regulation of gene expression is active on solid and in liquid media.

### Expression of full length polycistronic mRNAs

To confirm that all genes from the polycistronic mRNA were expressed with high efficiency only under inducing conditions, we aimed in semiquantitative RT-PCR analyses on the P2A_P2A transcript. Total RNA was isolated from liquid cultures containing the ATNT strain with the P2A_P2A construct. The cultures were grown for 24 h either without doxycycline or were induced for 6 or 24 h. cDNAs were generated with anchored oligo(dT) primers and cDNA levels from the different cultivation conditions were normalised against the *A. niger* actin gene. Oligonucleotides were deduced that amplify regions of the three individual genes that are contained in the polycistronic transcript (Fig. 3d). As shown in Fig. 3e, in the absence of doxycycline (Doxy 0 h) no amplification was observed on the 5′ and middle region of the polycistronic transcript encoding MelA and TyrP and only a weak band was detected for the gene sequence coding for tdTomato. This is in agreement with the low basal expression observed in *lacZ* reporter assays and in analysis of aspulvinone E metabolite production. However, strong induction was observed from both induced cultures (Doxy 6 h and Doxy 24 h) with PCR products on all three gene regions from the polycistronic messenger. This indicates that the full-length mRNA is efficiently transcribed only under doxycycline inducing conditions.

### Subcellular localisation of proteins

Colouration of the mycelium indicated functional production and separation of MelA and TyrP and transcript analyses showed that all three genes encoded on the single transcript were efficiently transcribed. However, these analyses did not confirm the correct separation of proteins from the second P2A peptide, which should result in a cytoplasmic localisation of tdTomato, nor confirmed the correct subcellular localisation of any of the proteins. Therefore, fluorescence microscopy analyses were performed. While the supplementation of liquid media with talc avoids pellet formation, it hampers microscopic analyses of hyphae due to the attached talc particles. On the other hand, individual hyphae are difficult to visualise once fungal pellets are formed. As our analyses showed that Asp-melanin is formed either on liquid or solid media, strains were grown on glucose minimal media containing agar plates and coverslips coated with doxycycline containing glucose minimal media were placed around the colonies. Once hyphae grew on the edges of the coverslips they were removed, embedded in a DAPI-containing mounting solution and analysed by fluorescence microscopy. As shown in Fig. 3f hyphae of the strain containing the single P2A separator, which means a fusion of the *tyrP* gene with the gene coding for the red fluorescent protein tdTomato, exclusively showed red fluorescence in subcellular organelles most likely resembling Golgi and ER [19]. This indicates that TyrP is quantitatively transported into the correct subcellular compartment after P2A cleavage from MelA and a functional TyrP-tdTomato fusion protein is produced. In contrast, hyphae of the strain containing the P2A_P2A construct showed very strong cytoplasmic red fluorescence, indicating that both P2A cleavage sites were correctly recognised and full length tdTomato had been produced. Observation of colonies grown in the absence of doxycycline only revealed DAPI staining of nuclei but no red fluorescence signal (not shown).

## Discussion

The aim of this study was the generation of an optimised fine-tuneable expression system in *A. niger* that produces high expression rates when fully induced and which is suitable to express polycistronic genes for recombinant expression of fungal secondary metabolite biosynthetic gene clusters. Previous studies investigated the suitability of *A. niger* as expression platform for the production of secondary metabolites such as the non-ribosomal peptide enniatin [33]. Thereby, the use of the Tet-on expression system combined with optimised fermentation conditions resulted in 4.5 g/l enniatin confirming both, the suitability of the Tet-on system to induce secondary metabolite production and the suitability of *A. niger* as expression platform. Similarly, we have previously shown that a range of different metabolites such as polyketides [18], non-ribosomal peptides [20] and products from NRPS-like enzymes [19] can be successfully produced in *A. niger*.

The combination of our TerR/P*terA*-system with the Tet-on-system resulted in an expression system with exceptionally high transcription rates which are still titratable. The coupling of Tet-on with the highly specific transcription factor TerR resulted in an amplification of gene expression by more than 5 times compared to direct gene expression from Tet-on. Therefore, even single copy integrations result in high transcript levels, which makes a selection for multi copy integration strains dispensable [17] and reduces adverse effects on growth and physiology caused by multiple random genome integration events. However, as our approach did not target a specific gene locus, some positioning effect from independent single copy integration strains was observed, which is resembled in the standard deviations from β-galactosidase activity determinations.

Another advantage of using the coupled Tet-on controlled TerR/P*terA* expression system compared to direct expression from the Tet-on promoter system is the low concentration of doxycycline required for induction of gene expression. Significant expression rates were already observed at 0.75 μg/ml doxycycline. This activity from the coupled system was similar to that obtained from the direct Tet-on controlled gene expression at 15 µg/ml. Low amounts of doxycycline reduce the risk of co-extraction of the inducer when aiming for purification of secondary metabolites. However, as highest expression rates were observed at 15 to 30 µg doxycycline, these should be used especially when producing toxic metabolites that require significant biomass production prior to high level induction of gene expression. As a proof-of-concept we showed that Asp-melanin is efficiently produced when strains were pre-grown for 18 h and induced by doxycycline for only 6 h.

The tight regulation of the Tet-on controlled TerR/P*terA* expression system combined with its high induction rate makes it also superior to our original P*amyB* controlled TerR/P*terA* expression system, which is constitutively active on sugar containing media [18]. As glucose containing medium is generally used in the regeneration of protoplasts in fungal transformations, the production of toxic natural products may prevent growth of positive transformants. In contrast, regeneration of protoplasts of ATNT16 strain in the absence of doxycycline prevents expression of heterologous genes. In this respect, when we expressed the aspulvinone E synthetase gene *melA* in the *A. niger* P2 strain (P*amyB* control of *terR* expression) we suffered from the reduced ability of fungal colonies to produce conidia [19]. In this study, ATNT16 *melA* ^OE^ strains grown in the absence of doxycycline were indistinguishable from the parental control strain, unless induced by doxycycline.

Importantly, the P2A polycistronic gene expression was compatible with our high level expression system. Asp-melanin was efficiently produced from both, the single P2A construct that contained the fusion of TyrP with tdTomato as well as the construct in which all three proteins were separated by P2A sequences. While we were not able to quantify the production of the insoluble pigment Asp-melanin, the brown colouration of the mycelium in the single P2A construct appeared less pronounced compared to the P2A_P2A construct (Fig. 3b, c). Positioning effects due to the order of genes in the polycistronic messenger as described previously [28, 30] that may in part be due to a drop-off of the ribosome after the translational skipping event [31] cannot account for this difference as the position of the *tyrP* gene was identical in both constructs. Therefore, it is likely that the fusion of TyrP with tdTomato affects activity of the tyrosinase. Nevertheless, this fusion unambiguously showed that the TyrP protein is correctly and quantitatively targeted to the ER and Golgi as (1) TyrP is inactive in the reducing environment of the cytoplasm [19] and therefore needs to be transported to the oxidative environment of Golgi and ER and (2) no cytoplasmic background fluorescence from tdTomato remaining in the cytoplasm was observed. Therefore, the recognition of the *N*-terminal subcellular localisation sequence has not been affected by the proline residue added to TyrP from the P2A peptide [24] when cleaved from the Aspulvinone E synthetase MelA. Whether *C*-terminal localisation sequences such as the peroxisomal PTS1 tripeptide import sequence SKL or AKL, as found in proteins of fungal siderophore biosynthesis [34], may be masked by the *C*-terminal addition of a P2A sequence needs to be tested in future studies.

This study also showed that the second P2A sequence in the P2A_P2A construct is efficiently cleaved as this construct showed extremely bright red fluorescence from the cytoplasm. However, due to the extremely bright fluorescence from this construct, which even leads to a reddish appearance of the edges of colonies on plates, we cannot exclude that some uncleaved protein may still be transported into the ER and Golgi. Nevertheless, due to the high fluorescence intensity from the cytoplasm combined with the high activity of the tyrosinase from the P2A_P2A construct, the majority of P2A peptides has efficiently been cleaved.

## Conclusion

The combination of tightly controlled Tet-on induction with the highly specific TerR/P*terA* expression system resulted in a well-regulated fine-tuneable and very strong gene expression. Therefore, the system appears suitable for high-level production of metabolites, even those with antifungal properties. In addition, the system is compatible with the use of self-cleaving peptides such as P2A. Cleavage sites are efficiently recognised and at least *N*-terminal secretion signals seem to remain unaffected. Therefore, this system can be used for the discovery of metabolites from yet unexplored fungal secondary metabolite biosynthetic gene clusters.

## Methods

### Media, fungal cultivation and transformation

Conidia suspensions were obtained by growing *Aspergillus niger* strains in slope cultures containing *Aspergillus* minimal medium with 50 mM glucose as carbon and 10 mM glutamine as nitrogen source [19] denoted as GG10 medium. For solid media 2% agar was added. Slopes were incubate for 4 days at 28 °C and overlaid with 6 ml phosphate-buffered saline (PBS) containing 0.01% Tween 20. Conidia were scraped into suspension using sterile cotton swaps. Suspensions were filtered over a 40 µm cell strainer (Greiner BioOne) to remove hyphae and clumps of conidia. After centrifugation the supernatant was discarded, conidia suspended in PBS and conidia concentrations determined using an improved Neubauer chamber. If not indicated otherwise, GG10 liquid cultures were inoculated with 1 × 10^6^ conidia/ml with or without the addition of doxycycline (final concentration 0–30 µg/ml) and incubated at 28 °C on a rotary shaker at 150 rpm. Mycelia and culture supernatants were separated by filtration over Miracloth filter gauze (Merck, Calbiochem). Mycelia were pressed dry between tissue paper and frozen in liquid nitrogen for subsequent analyses. Fungal transformation was performed as described previously [20] with some minor modifications. Mycelia for protoplast formation were generated by inoculating YEPD medium (20 g peptone, 10 g yeast extract, 5 g glucose per litre) with spores of the *A. niger* wild-type strain A1144 (Fungal Genetics Stock Center, Kansas, USA) or the expression platform strain ATNT16. After 22 h mycelia were washed and incubated for 60 min in 90 mM citrate–phosphate buffer pH 7.3 containing 10 mM dithiothreitol. Protoplasts were generated by using 1.3 g/20 ml sterile filtered VinoTaste Pro (Novozymes) in osmotic medium with 0.6 M KCl as osmotic stabiliser. After transformation protoplasts were regenerated on solid GG10 media containing 1.2 M sorbitol and either 40 µg/ml phleomycin, 140 µg/ml hygromycin B or 0.1 µg/ml pyrithiamine as selectable marker. Genomic DNA was isolated using the MasterPure Yeast DNA purification kit (Epicenter).

### Generation of the ATNT16 expression platform strain

All oligonucleotides used in this study are listed in Additional file 5: Table 1. All PCR reactions were performed in a SpeedCycler^2^ (Analytic Jena) in 10 µl volumes using either Phusion (fragment cloning, Thermo Scientific) or Phire Hot Start II DNA polymerase (colony PCR, Thermo Scientific) for DNA amplification. PCR fragments and digested plasmids for cloning purposes were gel purified using the GeneJet Gel Extraction Kit (Thermo Scientific). To generate the *A. niger* ATNT16 strain a plasmid was generated that contained the gene of the transcriptional activator *terR* under control of the Tet-on reverse transactivator system [22]. The construct was cloned into the *Hind*III linearized pUC19-ble [18] vector containing a phleomycin resistance cassette for selection of transformants. The Tet-on system was amplified with oligonucleotides 1 and 2 from plasmid pFW22.1 (kindly provided by V. Meyer, Berlin) and contained overhangs to the *Hind*III site of pUC19-ble. Subsequently, the *terR* gene including its own terminator sequence was amplified with oligonucleotides 3 and 4 from genomic DNA of *Aspergillus terreus* SBUG844 and cloned into the *Nco*I linearized TetOn_ble_pUC19 vector [12]. The 5′-end contained an overhang to the Tet-on system and at the 3′-end to the *Hind*III site of pUC19-ble. Linearized plasmid and gel-purified PCR products were mixed and assembled by in vitro recombination using the InFusion HD cloning kit (Takara/Clonetech) resulting in plasmid Tet-on*:terR*_ble_pUC19. The assembled plasmid was amplified in *Escherichia coli* DH5α using Mix & Go competent cells (Zymo Research). Positive clones were selected by colony PCR using oligonucleotides 5 and 6. Plasmids were isolated by use of the NucleoSpin Plasmid Miniprep kit (Macherey-Nagel) and correct assembly was confirmed by restriction analyses. The plasmid was used for transformation of *A. niger* A1144 and phleomycin resistant transformants were checked for single copy integration of the construct by Southern blot analysis using a dig labelled probe amplified with oligonucleotides 7 and 8. Transformant ATNT16 was selected for subsequent studies.

### Generation of *lacZ* reporter and aspulvinone E synthetase gene expressing strains

To generate the *lacZ* reporter strains in the ATNT16 background, plasmid *hph*_*tdTomato*:*lacZ*:*trpC* ^T^_pJET1.2 [18] containing the *lacZ* reporter under control of the *terA* promoter and a hygromycin B resistance cassette were used for transformation of ATNT16. A fusion of the Tet-on transactivator with the *lacZ* gene was assembled in the *Pst*I restricted pUC19_*ptrA* plasmid [35] containing the pyrithiamine resistance cassette as selectable marker. Tet-on was amplified from plasmid pFW22.1 with oligonucleotide 9 containing an overhang to the *Pst*I site of pUC19_*ptrA* and oligonucleotide 10. The *lacZ* gene including a *trpC* terminator was amplified from plasmid *hph*_*tdTomato*:*lacZ*:*trpC* ^T^_pJET1.2 with oligonucleotide 11 containing an overhang to the 3′-end of Tet-on and oligonucleotide 12 with overhang to the *Pst*I site of pUC19_*ptrA*. Linearized plasmid and the two gel-purified PCR fragments were mixed, assembled by in vitro recombination and transferred to *E. coli* as described above. Positive clones were selected by colony PCR using oligonucleotides 13 and 14. Isolated plasmids were checked by restriction analyses and used for transformation of A1144. Genomic DNA of ATNT16 and A1144 transformants was restricted with *Sma*I and analysed by Southern blot with a probe against the *lacZ* gene (oligonucleotides 13 and 14). At least three strains with a single copy integration of the reporter construct were used for expression analyses. For expression of the *A. terreus* aspulvinone E synthetase gene *melA* in the ATNT16 background, plasmid his_*melA*-SM-Xpress [19] was used as it contains a fusion of the *terA* promoter with the *melA* gene. The phleomycin resistance cassette of this plasmid was excised by *Not*I restriction and replaced by the pyrithiamine resistance cassette (*ptrA*) for transformation of ATNT16. Transformants were analysed by Southern blot with a probe against the *melA* gene (oligonucleotides 15 and 16) and strains with single copy integration were selected (Additional file 3).

### Generation of model gene cluster expressing strains

Two different polycistronic expression constructs were generated for gene expression in ATNT16. The first construct contained the *melA* gene and a fusion of the *tyrP* gene and the gene coding for tdTomato. The *melA* and *tyrP* genes were separated by a P2A coding sequence. The *melA* gene was amplified from genomic DNA of *A. terreus* SBUG844 with oligonucleotide 17 that contained an overlap to the *Nco*I restricted SM-Xpress2 vector [19] and oligonucleotide 18 with an overhang coding for a the P2A sequence. The gene fusion of *tyrP* with the tdTomato gene was amplified from plasmid *tyrP*:*tdTomato*_SM-Xpress2 [19] with oligonucleotide 19 possessing an overhang to the P2A sequence and oligonucleotide 20 with an overlap to the *Nco*I restricted SM-Xpress2 vector. In the second construct all three genes were separated by P2A sequences. The *melA* gene was amplified with the same oligonucleotides as for the first construct. The *tyrP* gene was amplified from genomic DNA of *A. terreus* SBUG844 with oligonucleotide 19 containing the P2A sequence overhang towards *melA* and oligonucleotide 21 with a P2A sequence overhang towards the tdTomato gene. Finally, the tdTomato gene was amplified from plasmid *tyrP*:*tdTomato*_SM-Xpress2 with oligonucleotide 22 possessing the complementary overhang to the *tyrP* 3′ P2A sequence and oligonucleotide 20 with a compatible overhang to the SM-Xpress2 vector. Constructs were assembled by in vitro recombination and transferred to *E. coli* DH5α. Clones were checked by colony PCR using oligonucleotides 23 and 24 to test for correct gene assembly. Plasmid DNA was isolated and used for transformation of ATNT16 with hygromycin B as selectable marker. Transformants were analysed by Southern blot with a probe against the *melA* gene (oligonucleotides 15 and 16) and strains with single copy integration were analysed further (Additional file 4).

### β-Galactosidase reporter assays

To study β-galactosidase reporter activities, fungi were inoculated at 2 × 10^6^ conidia per ml and grown for 24 h in 100 mM glucose containing minimal media with 20 mM glutamine as nitrogen source and 1% talc to avoid the formation of cell pellets. Mycelia were harvested over Miracloth, pressed dry and frozen in liquid nitrogen. Mycelia were ground to a fine powder under liquid nitrogen and suspended in Z buffer (60 mM Na_2_HPO_4_, 40 mM NaH_2_PO_4_, 10 mM KCl, 1 mM MgSO_4_, 0.7% β-mercaptoethanol). After centrifugation for 5 min at 16,000 × g and 4 °C the cell-free supernatant was removed and used for determination of β-galactosidase activity as previously described [36] using *o*-nitrophenyl-β-d-galactopyranoside (ONPG; ε = 3.5 mM^−1^ cm^−1^) as substrate. Protein concentrations were determined by using the Bradford Protein Assay (BioRad) with bovine serum albumin as standard. All spectrophotometric assays were carried out using an Evolution 220 UV–VIS spectrophotometer (ThermoFisher Scientific). From each construct three independent strains were grown in biological duplicates and activity determinations were made in technical duplicates.

### Analysis of aspulvinone E production

To test production of aspulvinone E in ATNT16 strains carrying a single copy integration of the P*terA*:*melA* construct, GG10 medium was inoculated with 1 × 10^6^ conidia/ml and one culture was supplemented with 15 µg/ml of doxycycline, whereas the other was left untreated. Incubation was performed for 48 h at 28 °C on a rotary shaker at 150 rpm. Mycelium was removed by filtration over Miracloth and the culture filtrate was extracted twice with an equal volume of ethyl acetate. After evaporation of the solvent under reduced pressure the residue was solved in 1 ml of methanol and subjected to HPLC analysis using a Dionex UltiMate3000 (ThermoFisher Scientific) and Eclipse XDB-C18 column, 5 μm, 4.6 × 150 mm (Agilent) that was kept at 40 °C. A binary solvent system consisting of water acidified with 0.1% formic acid (solvent A) and methanol (solvent B) was applied. The following gradient at a flow rate of 1 ml/min was used: 0–0.5 min 10% B, 0.5–15 min 10–90% B, 15–17 min 90% B, 17–17.5 min 90–100% B, 17.5–22 min 100% B, 22–23 min 100–10% B, 23–25 min 10% B. An authentic sample containing a mixture of aspulvinone E and isoaspulvinone E served as reference.

### Semiquantitative RT-PCR analyses

To analyse transcription of genes from the polycistronic messenger RNA of the ATNT16 P2A_P2A strain RNA was isolated using the MasterPure-Yeast RNA Purification Kit (Epicentre) from mycelium cultivated for 24 h in the absence or presence of 15 µg/ml doxycycline or pre-grown for 18 h without doxycycline and further cultivated for 6 h after addition of doxycycline. After a DNase treatment (TURBO DNase; ThermoFisher) RNA was transcribed into cDNA as previously described [19]. For normalisation of cDNA levels in the respective samples, serial dilutions were used for amplification of the *A. niger* actin gene using oligonucleotides 25 and 26. These primers span an intron region, which allows visualisation of a band shift from cDNA compared to genomic DNA (gDNA) and confirms the absence of contaminating gDNA in cDNA samples. For amplification of the *melA* gene oligonucleotides 15 and 16, for *tyrP* oligonucleotides 27 and 28 and for the tdTomato gene oligonucleotides 20 and 29 were used. PCRs of 30 cycles were performed in a SpeedCycler^2^ (Analytik Jena) using Phire Hot Start II polymerase (Thermo Scientific).

### Fluorescence microscopy

Fluorescence microscopy was performed as described previously [19] with some minor modifications. Strains were spotted on GG10 agar plates and pre-grown at 28 °C for one day, after which GG10 agarose coated coverslips containing 10 µg/ml doxycycline were placed next to the growing colony. 12 to 16 h later the coverslips were removed and placed on an object slide, overlaid with a droplet of mounting solution containing DAPI (ProLong Gold Antifade with DAPI, Thermo Scientific) and covered with a large coverslip. A GXML3201LED microscope (GX microscopes) was used for picture acquisition. Overlays of images were assembled by using the GIMP 2 software.

### Statistical analyses

Comparison of expression levels from β-galactosidase activity determinations were analysed using GraphPad Prism (GraphPad Software) by applying multiple t-tests using the Holm-Sidak method, with α = 0.05. Each row was analysed individually, without assuming a consistent standard deviation.

## Additional files

**Additional file 1.** Southern blot analyses and plasmid map of construct used for generation of ATNT strains. (**A**) Southern blot for identification of single copy integration strains. A digoxygenin labelled probe was used for hybridisation. Plasmid control and genomic DNA of parental strains and transformants were restricted with *Apa*I, which cuts once in the respective plasmid. The transformant used in subsequent analyses is numbered. (**B**) Plasmid map of the transformation construct. Position of oligonucleotides used in this study (P + number) as well as the position of the probe generated for Southern blot analysis and position of the restriction enzyme are shown. *ble* = phleomycin resistance cassette. TetOn = Tet-on promoter system. terR = *terR* gene including its native terminator sequence.

**Additional file 2.** Southern blot analyses and plasmid maps of constructs used for generation of *lacZ* reporter strains. (**A, C**) Southern blot for identification of single copy integration strains. Digoxygenin labelled probes were used for hybridisation. Transformants used in subsequent analyses are numbered. (**A**) A1144 strains with integration of the *tet*-*on:lacZ* construct. Plasmid control and genomic DNA of parental strains and transformants were restricted with *Ahd*I, which cuts once in the respective plasmid. (**C**) ATNT16 strain transformed with the P*terA:lacZ* construct. Plasmid control and genomic DNA of parental strains and transformants were restricted with *Hind*III, which cuts once in the respective plasmid. (**B, D**) Plasmid maps of the transformation constructs. Position of oligonucleotides used in this study (P + number) as well as the position of the probe generated for Southern blot analyses and position of the restriction enzyme are shown. ptrA = pyrithiamine resistance cassette. hph = hygromycin resistance casette. PterA = *terA* promoter from *Aspergillus terreus*. lacZ = β-galactosidase gene from *Escherichia coli*. TtrpC = *trpC* terminator sequence from *Aspergillus terreus.*

**Additional file 3.** Southern blot analysis and plasmid map of construct used for generation of ATNT *melA* strains. (**A**) Southern blot for identification of single copy integration strains. A digoxygenin labelled probe was used for hybridisation. Plasmid control and genomic DNA of parental strains and transformants were restricted with *Bgl*II, which cuts once in the respective plasmid. The transformant used in subsequent analyses is numbered. (**B**) Plasmid map of the transformation construct. Position of oligonucleotides used in this study (P + number) as well as the position of the probe generated for Southern blot analysis and position of the restriction enzyme are shown. ptrA = pyrithiamine resistance cassette. PterA = *terA* promoter from *Aspergillus terreus*. TtrpC = *trpC* terminator sequence from *Aspergillus terreus*. melA = Aspulvinone E synthetase gene *melA* from *Aspergillus terreus.*

**Additional file 4.** Southern blot analyses and plasmid maps of constructs used for generation of ATNT16 P2A_P2A and P2A strains. (**A**) Southern blot for identification of single copy integration strains. A digoxygenin labelled probe was used for hybridisation. Plasmid control and genomic DNA of parental strains and transformants were restricted with *Xba*I, which cuts once in the respective plasmids. Transformants used in subsequent analyses are numbered. (**B**, **C**) Plasmid maps of the transformation constructs. Position of oligonucleotides used in this study (P + number) as well as the position of the probe generated for Southern blot analysis and position of the restriction enzyme are shown. *hph* = hygromycin B resistance cassette. PterA = *terA* promoter from *Aspergillus terreus*. TtrpC = *trpC* terminator sequence from *Aspergillus terreus*. melA = Aspulvinone E synthetase gene *melA* from *Aspergillus terreus*. tyrP = tyrosinase gene *tyrP* from *Aspergillus terreus*. tdTomato = codon optimised *tdTomato* gene. tyrP:tdTom = fusion of *tyrP* and *tdTomato* genes. P2A = sequence coding for the 2A peptide from porcine teschovirus-1.

**Additional file 5: Table** **1.** Oligonucleotides used in this study.

## References

[1] Bok JW, Hoffmeister D, Maggio-Hall LA, Murillo R, Glasner JD, Keller NP (2006). Genomic mining for *Aspergillus* natural products. Chem Biol.

[2] Grigoriev IV, Nikitin R, Haridas S, Kuo A, Ohm R, Otillar R, Riley R, Salamov A, Zhao X, Korzeniewski F (2014). MycoCosm portal: gearing up for 1000 fungal genomes. Nucl Acids Res.

[3] de Vries RP, Riley R, Wiebenga A, Aguilar-Osorio G, Amillis S, Uchima CA, Anderluh G, Asadollahi M, Askin M, Barry K (2017). Comparative genomics reveals high biological diversity and specific adaptations in the industrially and medically important fungal genus *Aspergillus*. Genome Biol.

[4] Wasil Z, Pahirulzaman KAK, Butts C, Simpson TJ, Lazarus CM, Cox RJ (2013). One pathway, many compounds: heterologous expression of a fungal biosynthetic pathway reveals its intrinsic potential for diversity. Chem Sci.

[5] Gressler M, Meyer F, Heine D, Hortschansky P, Hertweck C, Brock M (2015). Phytotoxin production in *Aspergillus terreus* is regulated by independent environmental signals. eLife.

[6] Alberti F, Foster GD, Bailey AM (2017). Natural products from filamentous fungi and production by heterologous expression. Appl Microbiol Biotechnol.

[7] Anyaogu DC, Mortensen UH (2015). Heterologous production of fungal secondary metabolites in Aspergilli. Front Microbiol.

[8] Gonzalez-Menendez V, Perez-Bonilla M, Perez-Victoria I, Martin J, Munoz F, Reyes F, Tormo JR, Genilloud O (2016). Multicomponent analysis of the differential induction of secondary metabolite profiles in fungal endophytes. Molecules.

[9] Netzker T, Fischer J, Weber J, Mattern DJ, Konig CC, Valiante V, Schroeckh V, Brakhage AA (2015). Microbial communication leading to the activation of silent fungal secondary metabolite gene clusters. Front Microbiol.

[10] Brakhage AA, Schroeckh V (2011). Fungal secondary metabolites–strategies to activate silent gene clusters. Fungal Genet Biol.

[11] Brakhage AA (2013). Regulation of fungal secondary metabolism. Nat Rev Microbiol.

[12] Gressler M, Zaehle C, Scherlach K, Hertweck C, Brock M (2011). Multifactorial induction of an orphan PKS-NRPS gene cluster in *Aspergillus terreus*. Chem Biol.

[13] Clevenger KD, Bok JW, Ye R, Miley GP, Verdan MH, Velk T, Chen C, Yang K, Robey MT, Gao P (2017). A scalable platform to identify fungal secondary metabolites and their gene clusters. Nat Chem Biol.

[14] Bok JW, Keller NP (2004). LaeA, a regulator of secondary metabolism in *Aspergillus* spp. Eukaryot Cell.

[15] Sakai K, Kinoshita H, Nihira T (2012). Heterologous expression system in *Aspergillus oryzae* for fungal biosynthetic gene clusters of secondary metabolites. Appl Microbiol Biotechnol.

[16] Yeh HH, Ahuja M, Chiang YM, Oakley CE, Moore S, Yoon O, Hajovsky H, Bok JW, Keller NP, Wang CC, Oakley BR (2016). Resistance gene-guided genome mining: serial promoter exchanges in *Aspergillus nidulans* reveal the biosynthetic pathway for fellutamide B, a proteasome inhibitor. ACS Chem Biol.

[17] Fleissner A, Dersch P (2010). Expression and export: recombinant protein production systems for *Aspergillus*. Appl Microbiol Biotechnol.

[18] Gressler M, Hortschansky P, Geib E, Brock M (2015). A new high-performance heterologous fungal expression system based on regulatory elements from the *Aspergillus terreus* terrein gene cluster. Front Microbiol.

[19] Geib E, Gressler M, Viediernikova I, Hillmann F, Jacobsen ID, Nietzsche S, Hertweck C, Brock M (2016). A non-canonical melanin biosynthesis pathway protects *Aspergillus terreus* conidia from environmental stress. Cell Chem Biol.

[20] Brandenburger E, Gressler M, Leonhardt R, Lackner G, Habel A, Hertweck C, Brock M, Hoffmeister D (2017). A highly conserved basidiomycete peptide synthetase produces a trimeric hydroxamate siderophore. Appl Environ Microbiol.

[21] Meyer V, Wanka F, van Gent J, Arentshorst M, van den Hondel CA, Ram AF (2011). Fungal gene expression on demand: an inducible, tunable, and metabolism-independent expression system for *Aspergillus niger*. Appl Environ Microbiol.

[22] Wanka F, Cairns T, Boecker S, Berens C, Happel A, Zheng X, Sun J, Krappmann S, Meyer V (2016). Tet-on, or Tet-off, that is the question: advanced conditional gene expression in *Aspergillus*. Fungal Genet Biol.

[23] Tang X, Liu X, Tao G, Qin M, Yin G, Suo J, Suo X (2016). “Self-cleaving” 2A peptide from porcine teschovirus-1 mediates cleavage of dual fluorescent proteins in transgenic *Eimeria tenella*. Vet Res.

[24] Kim JH, Lee SR, Li LH, Park HJ, Park JH, Lee KY, Kim MK, Shin BA, Choi SY (2011). High cleavage efficiency of a 2A peptide derived from porcine teschovirus-1 in human cell lines, zebrafish and mice. PLoS One.

[25] Beekwilder J, van Rossum HM, Koopman F, Sonntag F, Buchhaupt M, Schrader J, Hall RD, Bosch D, Pronk JT, van Maris AJ, Daran JM (2014). Polycistronic expression of a beta-carotene biosynthetic pathway in *Saccharomyces cerevisiae* coupled to beta-ionone production. J Biotechnol.

[26] Efimova VS, Isaeva LV, Makeeva DS, Rubtsov MA, Novikova LA (2017). Expression of cholesterol hydroxylase/lyase system proteins in yeast *S. cerevisiae* cells as a self-processing polyprotein. Mol Biotechnol.

[27] de Amorim Araujo J, Ferreira TC, Rubini MR, Duran AG, De Marco JL, de Moraes LM, Torres FA (2015). Coexpression of cellulases in *Pichia pastoris* as a self-processing protein fusion. AMB Express.

[28] Subramanian V, Schuster LA, Moore KT, Taylor LE, Baker JO, Vander Wall TA, Linger JG, Himmel ME, Decker SR (2017). A versatile 2A peptide-based bicistronic protein expressing platform for the industrial cellulase producing fungus, *Trichoderma reesei*. Biotechnol Biofuels.

[29] Unkles SE, Valiante V, Mattern DJ, Brakhage AA (2014). Synthetic biology tools for bioprospecting of natural products in eukaryotes. Chem Biol.

[30] Schuetze T, Meyer V (2017). Polycistronic gene expression in *Aspergillus niger*. Microb Cell Fact.

[31] Liu Z, Chen O, Wall JBJ, Zheng M, Zhou Y, Wang L, Ruth Vaseghi H, Qian L, Liu J (2017). Systematic comparison of 2A peptides for cloning multi-genes in a polycistronic vector. Sci Rep.

[32] Driouch H, Sommer B, Wittmann C (2010). Morphology engineering of *Aspergillus niger* for improved enzyme production. Biotechnol Bioeng.

[33] Richter L, Wanka F, Boecker S, Storm D, Kurt T, Vural O, Sussmuth R, Meyer V (2014). Engineering of *Aspergillus niger* for the production of secondary metabolites. Fungal Biol Biotechnol.

[34] Grundlinger M, Yasmin S, Lechner BE, Geley S, Schrettl M, Hynes M, Haas H (2013). Fungal siderophore biosynthesis is partially localized in peroxisomes. Mol Microbiol.

[35] Fleck CB, Brock M (2010). *Aspergillus fumigatus* catalytic glucokinase and hexokinase: expression analysis and importance for germination, growth, and conidiation. Eukaryot Cell.

[36] Ebel F, Schwienbacher M, Beyer J, Heesemann J, Brakhage AA, Brock M (2006). Analysis of the regulation, expression, and localisation of the isocitrate lyase from *Aspergillus fumigatus*, a potential target for antifungal drug development. Fungal Genet Biol.

