# Supplementary material for: ATNT: an enhanced system for expression of polycistronic secondary metabolite gene clusters in Aspergillus niger
Source: Fungal Biol Biotechnol. 2017 Dec 19;4:13. doi: 10.1186/s40694-017-0042-1 (PMC5735947; doi:10.1186/s40694-017-0042-1)
Supplement: Supplementary file 1 — Additional file 1. Southern blot analyses and plasmid map of construct used for generation of ATNT strains. (A) Southern blot for identification of single copy integration strains. A digoxygenin labelled probe was used for hybridisation. Plasmid control and genomic DNA of parental strains and transformants were restricted with ApaI, which cuts once in the respective plasmid. The transformant used in subsequent analyses is numbered. (B) Plasmid map of the transformation construct. Position of oligonucleotides used in this study (P + number) as well as the position of the probe generated for Southern blot analysis and position of the restriction enzyme are shown. ble = phleomycin resistance cassette. TetOn = Tet-on promoter system. terR = terR gene including its native terminator sequence. [file 40694_2017_42_MOESM1_ESM.pdf]

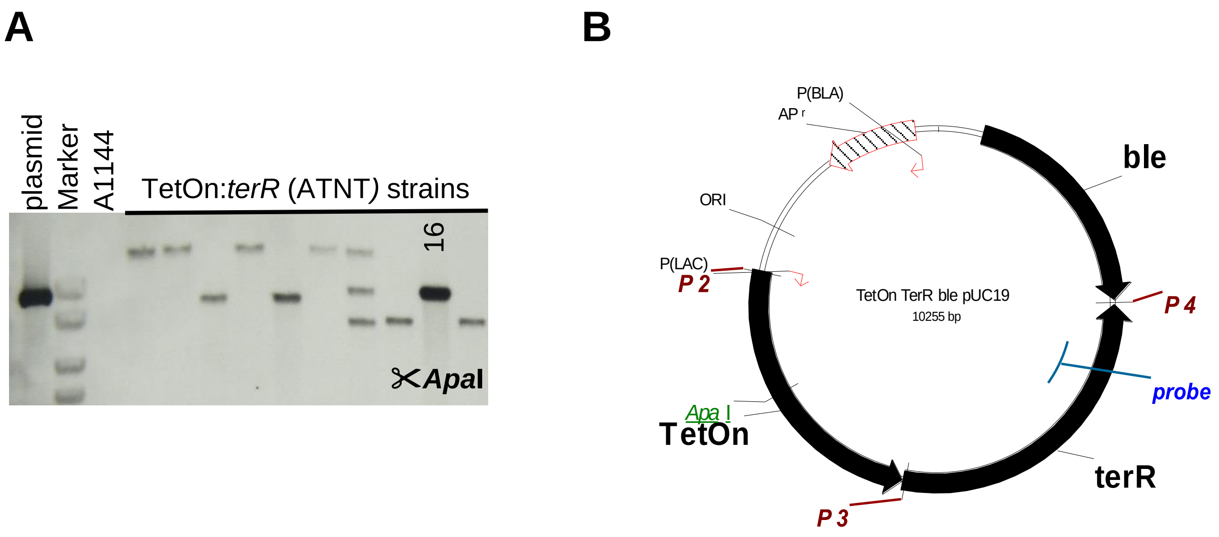

Additional file 1: Southern blot analyses and plasmid map of construct used for generation of ATNT strains. (**A**) Southern blot for identification of single copy integration strains. A digoxygenin labelled probe was used for hybridisation. Plasmid control and genomic DNA of parental strains and transformants were restricted with *Apa*I, which cuts once in the respective plasmid. The transformant used in subsequent analyses is numbered. (**B**) Plasmid map of the transformation construct. Position of oligonucleotides used in this study (P + number) as well as the position of the probe generated for Southern blot analysis and position of the restriction enzyme are shown. *ble* = phleomycin resistance cassette. TetOn = Tet-on promoter system. terR = *terR* gene including its native terminator sequence.
