# Supplementary material for: ATNT: an enhanced system for expression of polycistronic secondary metabolite gene clusters in Aspergillus niger
Source: Fungal Biol Biotechnol. 2017 Dec 19;4:13. doi: 10.1186/s40694-017-0042-1 (PMC5735947; doi:10.1186/s40694-017-0042-1)
Supplement: Supplementary file 2 — Additional file 2. Southern blot analyses and plasmid maps of constructs used for generation of lacZ reporter strains. (A, C) Southern blot for identification of single copy integration strains. Digoxygenin labelled probes were used for hybridisation. Transformants used in subsequent analyses are numbered. (A) A1144 strains with integration of the tet-on:lacZ construct. Plasmid control and genomic DNA of parental strains and transformants were restricted with AhdI, which cuts once in the respective plasmid. (C) ATNT16 strain transformed with the PterA:lacZ construct. Plasmid control and genomic DNA of parental strains and transformants were restricted with HindIII, which cuts once in the respective plasmid. (B, D) Plasmid maps of the transformation constructs. Position of oligonucleotides used in this study (P + number) as well as the position of the probe generated for Southern blot analyses and position of the restriction enzyme are shown. ptrA = pyrithiamine resistance cassette. hph = hygromycin resistance casette. PterA = terA promoter from Aspergillus terreus. lacZ = β-galactosidase gene from Escherichia coli. TtrpC = trpC terminator sequence from Aspergillus terreus. [file 40694_2017_42_MOESM2_ESM.pdf]

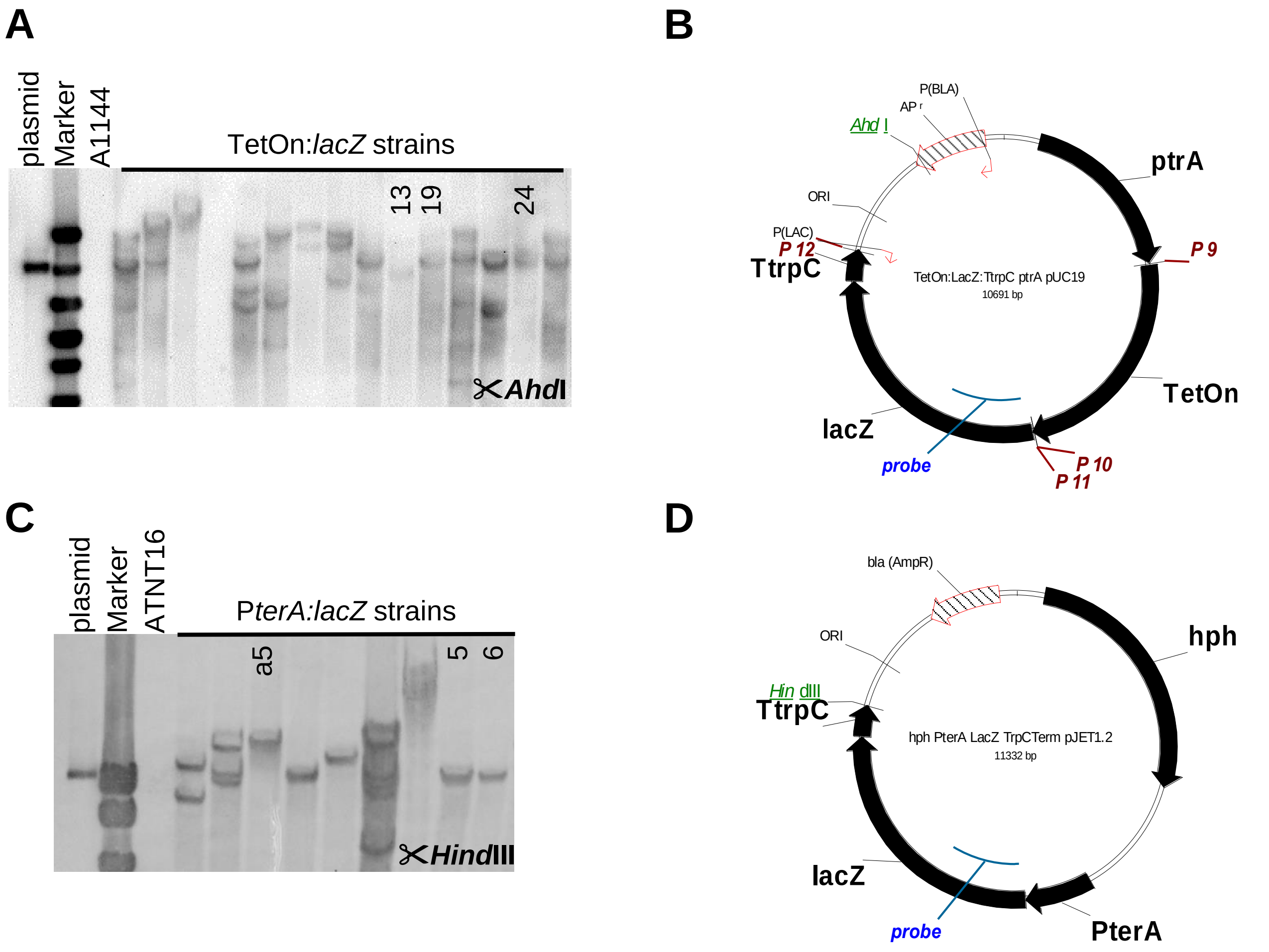

Additional file 2: Southern blot analyses and plasmid maps of constructs used for generation of *lacZ* reporter strains. (**A, C**) Southern blot for identification of single copy integration strains. Digoxygenin labelled probes were used for hybridisation. Transformants used in subsequent analyses are numbered. (**A**) A1144 strains with integration of the *tet-on:lacZ* construct. Plasmid control and genomic DNA of parental strains and transformants were restricted with *Ahd*I, which cuts once in the respective plasmid. (**C**) ATNT16 strain transformed with the P*terA:lacZ* construct. Plasmid control and genomic DNA of parental strains and transformants were restricted with *Hind*III, which cuts once in the respective plasmid. (**B, D**) Plasmid maps of the transformation constructs. Position of oligonucleotides used in this study (P + number) as well as the position of the probe generated for Southern blot analyses and position of the restriction enzyme are shown. ptrA = pyrithiamine resistance cassette. hph = hygromycin resistance casette. PterA = *terA* promoter from *Aspergillus terreus*. lacZ = β-galactosidase gene from *Escherichia coli*. TtrpC = *trpC* terminator sequence from *Aspergillus terreus*.
