# Supplementary material for: ATNT: an enhanced system for expression of polycistronic secondary metabolite gene clusters in Aspergillus niger
Source: Fungal Biol Biotechnol. 2017 Dec 19;4:13. doi: 10.1186/s40694-017-0042-1 (PMC5735947; doi:10.1186/s40694-017-0042-1)
Supplement: Supplementary file 3 — Additional file 3. Southern blot analysis and plasmid map of construct used for generation of ATNT melA strains. (A) Southern blot for identification of single copy integration strains. A digoxygenin labelled probe was used for hybridisation. Plasmid control and genomic DNA of parental strains and transformants were restricted with BglII, which cuts once in the respective plasmid. The transformant used in subsequent analyses is numbered. (B) Plasmid map of the transformation construct. Position of oligonucleotides used in this study (P + number) as well as the position of the probe generated for Southern blot analysis and position of the restriction enzyme are shown. ptrA = pyrithiamine resistance cassette. PterA = terA promoter from Aspergillus terreus. TtrpC = trpC terminator sequence from Aspergillus terreus. melA = Aspulvinone E synthetase gene melA from Aspergillus terreus. [file 40694_2017_42_MOESM3_ESM.pdf]

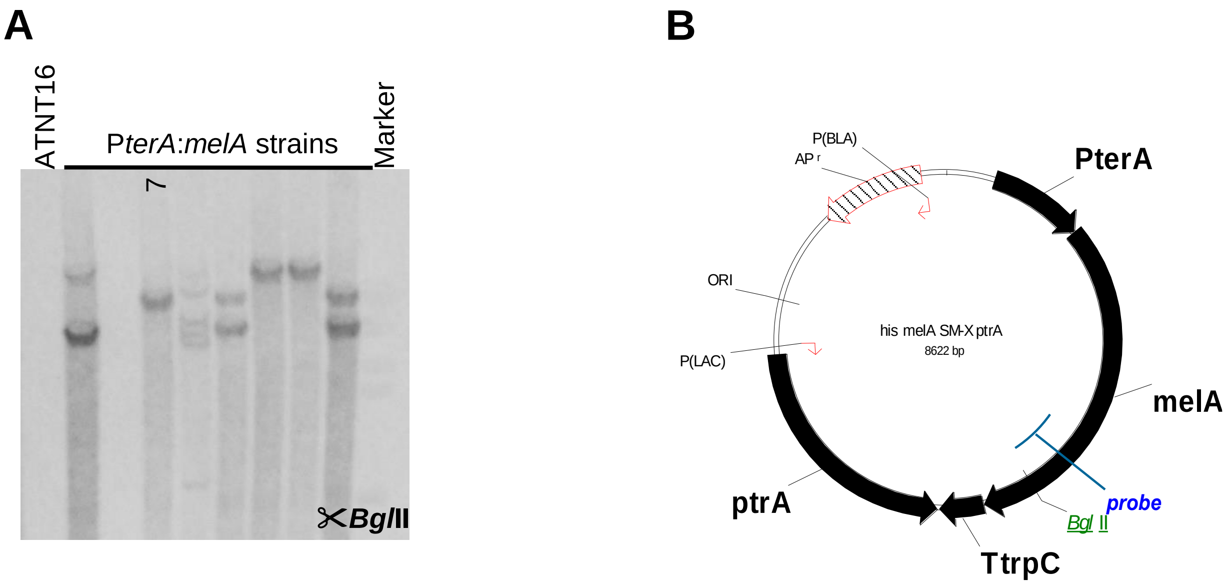

Additional file 3: Southern blot analysis and plasmid map of construct used for generation of ATNT *melA* strains. (**A**) Southern blot for identification of single copy integration strains. A digoxygenin labelled probe was used for hybridisation. Plasmid control and genomic DNA of parental strains and transformants were restricted with *Bgl*II, which cuts once in the respective plasmid. The transformant used in subsequent analyses is numbered. (**B**) Plasmid map of the transformation construct. Position of oligonucleotides used in this study (P + number) as well as the position of the probe generated for Southern blot analysis and position of the restriction enzyme are shown. ptrA = pyrithiamine resistance cassette. PterA = *terA* promoter from *Aspergillus terreus*. TtrpC = *trpC* terminator sequence from *Aspergillus terreus*. melA = Aspulvinone E-synthetase gene *melA* from *Aspergillus terreus*.
