# Supplementary material for: ATNT: an enhanced system for expression of polycistronic secondary metabolite gene clusters in Aspergillus niger
Source: Fungal Biol Biotechnol. 2017 Dec 19;4:13. doi: 10.1186/s40694-017-0042-1 (PMC5735947; doi:10.1186/s40694-017-0042-1)
Supplement: Supplementary file 4 — Additional file 4. Southern blot analyses and plasmid maps of constructs used for generation of ATNT16 P2A_P2A and P2A strains. (A) Southern blot for identification of single copy integration strains. A digoxygenin labelled probe was used for hybridisation. Plasmid control and genomic DNA of parental strains and transformants were restricted with XbaI, which cuts once in the respective plasmids. Transformants used in subsequent analyses are numbered. (B, C) Plasmid maps of the transformation constructs. Position of oligonucleotides used in this study (P + number) as well as the position of the probe generated for Southern blot analysis and position of the restriction enzyme are shown. hph = hygromycin B resistance cassette. PterA = terA promoter from Aspergillus terreus. TtrpC = trpC terminator sequence from Aspergillus terreus. melA = Aspulvinone E synthetase gene melA from Aspergillus terreus. tyrP = tyrosinase gene tyrP from Aspergillus terreus. tdTomato = codon optimised tdTomato gene. tyrP:tdTom = fusion of tyrP and tdTomato genes. P2A = sequence coding for the 2A peptide from porcine teschovirus-1. [file 40694_2017_42_MOESM4_ESM.pdf]

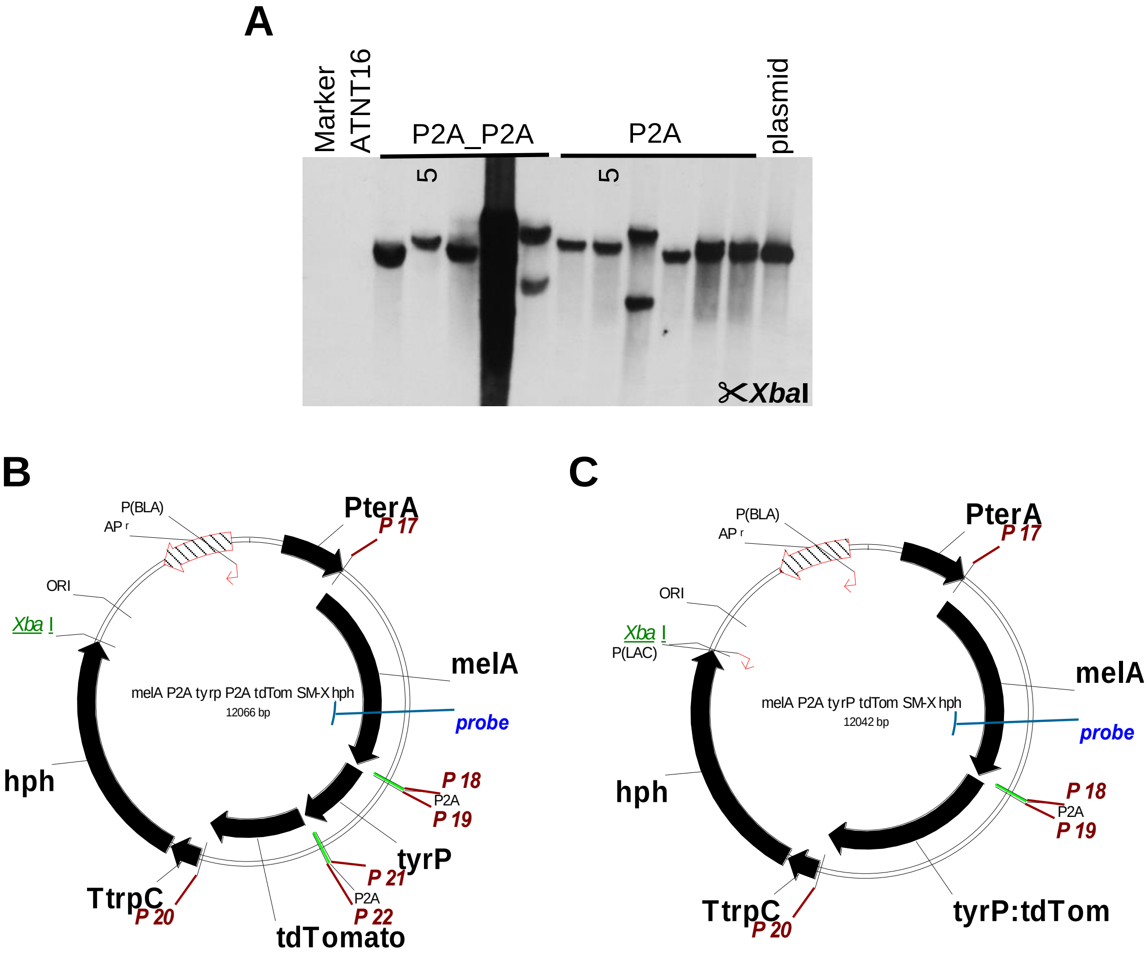

Additional file 4: Southern blot analyses and plasmid maps of constructs used for generation of ATNT16 P2A_P2A and P2A strains. (**A**) Southern blot for identification of single copy integration strains. A digoxygenin labelled probe was used for hybridisation. Plasmid control and genomic DNA of parental strains and transformants were restricted with *Xba*I, which cuts once in the respective plasmids. Transformants used in subsequent analyses are numbered. (**B**, **C**) Plasmid maps of the transformation constructs. Position of oligonucleotides used in this study (P + number) as well as the position of the probe generated for Southern blot analysis and position of the restriction enzyme are shown. *hph* = hygromycin B resistance cassette. PterA = *terA* promoter from *Aspergillus terreus*. TtrpC = *trpC* terminator sequence from *Aspergillus terreus*. melA = Aspulvinone E-synthetase gene *melA* from *Aspergillus terreus*. tyrP = tyrosinase gene *tyrP* from *Aspergillus terreus*. td-Tomato = codon optimised *td-Tomato* gene. tyrP:td-Tom = fusion of *tyrP* and *td-Tomato* genes. P2A = sequence coding for the 2A peptide from porcine teschovirus-1.
