# Supplementary material for: ATNT: an enhanced system for expression of polycistronic secondary metabolite gene clusters in Aspergillus niger
Source: Fungal Biol Biotechnol. 2017 Dec 19;4:13. doi: 10.1186/s40694-017-0042-1 (PMC5735947; doi:10.1186/s40694-017-0042-1)
Supplement: Supplementary file 5 — Additional file 5: Table 1. Oligonucleotides used in this study. [file 40694_2017_42_MOESM5_ESM.docx]

**Table 1: Oligonucleotides used in this study.**

| **#** | **Sequence** | **Feature** |
| --- | --- | --- |
| **1** | CGACCTGCAGGCATGCAAGCTTCCATGGTGTTTAAACGGTGATGTCT | TetOn_*ble*_pUC19 |
| **2** | TGACCATGATTACGCCAAGCTTCCCTCGGCTGGTC | TetOn*_ble_*pUC19 |
| **3** | CACCGTTTAAACACCATGTTCGCCGAACTTAACGCAAAG | TetOn*:terR_ble*_pUC19 |
| **4** | GACCTGCAGGCATGCAAGCTTCGCATCACGTCTTGTTATCTGTTG | TetOn*:terR_ble*_pUC19 |
| **5** | CAAGAAGAAGCATGTGGTACC | control primer *terR* |
| **6** | GATGGCCAACACAGCTTGG | control primer *terR* |
| **7** | GTCAGCATGCCAACGCTCTC | probe primer *terR* |
| **8** | CCAAGAAAGTCACACAGATCG | probe primer *terR* |
| **9** | TCCTCTAGAGTCGACCTGCAGCCCTCGGCTGGTCTGTCTTAC | TetOn*:lacZ_ptrA*_pUC19 |
| **10** | GGTGATGTCTGCTCAAGCG | TetOn*:lacZ_ptrA*_pUC19 |
| **11** | TGAGCAGACATCACCATGAGATCCACCATGATTACGG | TetOn*:lacZ_ptrA*_pUC19 |
| **12** | CCAAGCTTGCATGCCTGCAGGAGTGAGGGTTGAGTACGAG | TetOn*:lacZ_ptrA*_pUC19 |
| **13** | GGCGTTACCCAACTTAATCGC | control primer *lacZ* |
| **14** | CTCATCCATGACCTGACCATG | control primer *lacZ* |
| **15** | CGACGTTGGCATTGACTCC | probe primer *melA* |
| **16** | GCGCATCTTAATGTGTGGAGG | probe primer *melA* |
| **17** | CATCACAGCACCATGCATCACCATCACCATCACCAACCAAGCCTTATTCCCTC | his_melA_P2A_XXX_SM-Xpress |
| **18** | CCGGCTTGCTTCAGGAGGCTGAAATTGGTAGCGCCGCTGCCCATGCCCCTCTCAGCAAGAG | P2A constructs |
| **19** | TCCTGAAGCAAGCCGGTGATGTGGAGGAAAACCCTGGCCCTATGGGTTTCTACAGGAATCTAG | P2A constructs |
| **20** | ATCACTGCTGCCATGCTACTTGTAGAGCTCGTCCATAC | P2A constructs |
| **21** | CAGCCTGTTTGAGCAGGGAAAAGTTAGTGGCACCGGAACCGGTATATGTATAACAATAAGGACC | P2A constructs |
| **22** | CTGCTCAAACAGGCTGGCGACGTCGAAGAGAATCCCGGTCCCATGGTCTCCAAGGGTGAGG | P2A constructs |
| **23** | ACGGACCATACGCTCTCGCCGGCTATTCCTATGGG | colony PCR P2A constructs |
| **24** | GAAAGGGACGTTTCGGTGC | colony PCR P2A constructs |
| **25** | GCGAGAGGAGAAAGACTGG | RT-PCR actin |
| **26** | CAAGCCAGCAAGAATACCACC | RT-PCR actin |
| **27** | CCCATGATATTCTGAAATTCG | RT-PCR *tyrP* |
| **28** | CCTCCTGAGCTATCAGTCG | RT-PCR *tryP* |
| **29** | CCAAGCTCGACATCACCTCC | RT-PCR *tdTom* |
